# Explainability in AI-enabled medical neurotechnology: a scoping review

**DOI:** 10.1186/s12984-026-01892-0

**Published:** 2026-02-04

**Authors:** Laura Schopp, Georg Starke, Marcello Ienca

**Affiliations:** 1 Institute of History and Ethics in Medicine, School of Medicine and Health, Technical University of Munich, Munich, Germany; 2 Freiburg Institute for Advanced Studies, University of Freiburg, Freiburg im Breisgau, Germany; 3 Munich Institute for Robotics and Machine Intelligence (MIRMI), Technical University of Munich (TUM), Munich, Germany

**Keywords:** Explainable artificial intelligence, Neurotechnology, Brain-computer interface, Neurostimulation, Neurorehabilitation, Neurological disease, Psychiatric disorder

## Abstract

Artificial Intelligence (AI) approaches, including Machine Learning (ML), and other complex algorithms, are driving progress in medical closed-loop neurotechnology, including neurostimulation systems and brain–computer interfaces (BCIs). These advances are transforming the treatment landscape for neurological and psychiatric conditions. However, the inherent opacity of many AI models raises clinical, epistemological and ethical challenges. Explainability is widely recognized as a critical requirement for addressing these challenges, yet its concrete application in neurotechnology remains insufficiently explored. *Objective.* This scoping review maps how Explainable AI (XAI) methods are implemented in AI-enabled closed-loop neurotechnologies and examines how explainability is conceptualized and operationalized in this domain. *Approach.* Following JBI guidance and PRISMA-ScR, we systematically searched five databases for original research on AI-enabled medical closed-loop neurotechnologies targeting neurological and psychiatric conditions. A total of 161 studies were included and analyzed using descriptive statistics and qualitative content analysis to identify the presence and framing of XAI methods. *Main results*. Explainable AI adoption in medical neurotechnology is limited: only 14 studies (9%) employed explicit XAI techniques. Thematic analysis of the full corpus identified three recurring barriers and focal points: (A) technical constraints and proposed workarounds, (B) challenges in explanation quality and system transparency, and (C) the relationship between explainability and user trust. *Significance.* Although closed-loop neurotechnologies are rapidly advancing, explainability is rarely implemented in practice, constraining transparency, accountability, and clinical usability. Our findings reveal key factors behind the explainability gap and provides a framework to guide future research and development. Addressing this shortfall is essential for fostering ethically sound, clinically effective, and patient-trusted neurotechnological applications.

## Introduction

 The opacity of Artificial Intelligence (AI) systems in healthcare raises major ethical and legal challenges and poses a primary obstacle to the broader adoption of AI in healthcare. AI opacity refers to the lack of transparency in how AI models process data and generate outputs. This can result from the complexity of the model itself (e.g., deep neural networks with millions of parameters), the proprietary nature of algorithms, or the absence of clear documentation on decision-making processes. Opaque AI systems are thought to undermine respect for patient autonomy, hinder accountability, and impede user trust [1, 2]. Offering measures to render AI systems more intelligible to humans in clinical settings is therefore widely accepted as crucial [3, 4], both to increase technology acceptance among healthcare professionals as well as to comply with legal demands such as the transparency requirements enshrined in Article 13 of the European Union (EU) AI Act [5, 6].

To mitigate opacity-related challenges of AI in healthcare, Explainable AI (XAI) has emerged as a widely-heralded solution [7–10]. When applied and interpreted correctly, XAI methods can offer deeper insights into an algorithm’s underlying processes by transforming an otherwise non-interpretable model into one that provides understandable insights [11]. Frequently, such explanations take the shape of post-hoc assessments of ‘feature importance’ or ‘feature relevance’ –two terms often used interchangeably– to describe the contribution of individual features to a model’s output [12]. However, XAI encompasses a diverse range of approaches, incorporating various computational and visualization techniques, some of which are tailored to specific AI models [12–14].

In medical fields such as dermatology [15], radiology [16], oncology [17, 18], and gynecology [19], XAI methods have already been successfully implemented, enhancing the interpretability of AI-enabled diagnostics and decision-making processes. XAI approaches are increasingly being applied in neurology [20, 21] and psychiatry [22], particularly in brain abnormality diagnostics and mental health applications, where AI assists with tasks such as identifying subtle and complex patterns within large, heterogeneous neuroimaging datasets or powering mental health chatbots based on LargeLanguage Models (LLMs). In recent years, AI has become central also to clinical neurotechnologies such as Brain-Computer Interfaces (BCIs) and neurostimulation [23]. BCIs, neurostimulation systems such as Deep Brain Stimulation (DBS), and other neurotechnologies increasingly rely on machine learning (ML) and especially deep learning (DL) models to improve accuracy and performance [24]. For example, AI algorithms enhance BCIs by improving neural signal interpretation and mitigating noise in EEG time series data [24]. In DBS, DL has been leveraged to assist with image-guided surgical planning, optimizing electrode placement and treatment efficacy [25]. A growing proportion of clinical neurotechnologies today operate using a closed-loop (hereafter CL) architecture, in which systems continuously sense neural or physiological activity and automatically adjust stimulation or decoding strategies in response to changing brain states [26]. This stands in contrast to traditional open-loop systems, where parameters are set manually and remain fixed between clinical visits. CL neurotechnologies aim to improve efficacy, reduce side effects, and enable individualized, biomarker-driven, state-contingent, and energy-efficient interventions [27]. In this review, we focus on CL neurotechnologies because they raise a distinct set of epistemic, clinical and ethical considerations. Their adaptive and partially autonomous behavior increases the complexity and opacity of device function [28], making questions of explainability, clinical oversight, and patient autonomy particularly acute. Although open-loop devices are currently more widespread in clinical practice [29], the ongoing shift toward adaptive systems in epilepsy, movement disorders, and psychiatric indications makes CL neurotechnologies a critical test case for explainable AI in the clinical neuroscience domain. Given that these technologies interact directly with the human brain, ensuring their transparency and trustworthiness is especially critical not only to foster clinical acceptance but also to protect patient autonomy and safety.

Despite the growing need for explainability in medical CL neurotechnology, little is known about the extent to which XAI methods are currently employed or discussed in this domain. Our review addresses this gap by examining the prevalence of XAI approaches in medical CL neurotechnology. Previous studies have either broadly explored XAI in neurology [30] and psychiatry [22, 30, 31] without specifically addressing medical CL neurotechnology or relied exclusively on explainability-related keywords [30] thus overlooking the fact that XAI methods applied in medical CL neurotechnology may not explicitly self-identify as such. Furthermore, they have not further investigated the factors that facilitate or hinder adoption. In contrast, our review takes a more comprehensive and unbiased approach. Rather than restricting our analysis to studies that explicitly use XAI terminology, we conduct a review of all research applying AI or other complex algorithmic methods to CL neurotechnology. We then assess how many of these studies incorporate explainable methods, regardless of whether they are explicitly labeled as such. This methodology allows us to more accurately map the landscape of XAI applications in medical CL neurotechnology, measure their prevalence, and avoid selection bias that could arise from relying solely on self-reported explainability claims. By taking this broader perspective, our review provides a more representative picture of the role that explainability plays in medical CL neurotechnology today. Furthermore, we identify key factors influencing the adoption of XAI in medical CL neurotechnology and highlight the limitations that may hinder its broader implementation. Our findings emphasize the need for further interdisciplinary research and stakeholder engagement to ensure that future regulatory standards adequately support medical professionals. As this analysis attests, fostering explainability in AI-enabled medical CL neurotechnologies is essential to ensure that these systems serve both clinical needs and patient well-being effectively.

## Methods

To identify the current state of explainability in AI and other complex algorithm-based approaches to neurotechnologies, we conducted a scoping review using systematic and reproducible search methods, following JBI guidance and reported according to PRISMA-ScR. The focus was on articles using neurotechnologies that used AI and other complex algorithms for medical purposes. Specifically, our search was designed to identify research on medical CL neurotechnology that uses complex algorithms in relevant medical domains. As our aim was to map the field rather than evaluate intervention effectiveness, no formal risk-of-bias assessment was performed, consistent with scoping-review methodology [34].

The search was performed on January 12, 2025 across five databases (ACM, ArXiv, IEEE Xplore, PubMed, Scopus) using the following search heuristic logic:

- ((“brain-computer interface” OR neurostimulat* OR “neural prosthe*” OR neuroprosthe* OR neurotechnolog* OR neuromodulat* OR “deep brain stimulat*” OR “cortex stimulation”) AND.
- (“machine learning” OR “artificial intelligence” OR a.i. OR “deep learning” OR “closed-loop” OR adaptive OR responsive OR automat*) AND.
- (psychiatr* OR neurolo* OR clinical OR neuropsychiatr* OR neurorehab*))

in title, abstract, and keywords or similar individual database filters.

The search strategy included this latter clinical domain filter to reflects the review’s intentional focus on neurotechnology applications in clinical neurology and psychiatry, where CL systems directly interface with or modulate the nervous system. We deliberately selected our keywords to enable a comprehensive assessment of explainable AI without being limited by its terminological ambiguity. By omitting “explainability” as a direct search term, we acknowledged the semantic variability in the field and instead adopted an inductive approach to identify XAI methods and assess their relevance during the full-text analysis phase.

**Fig. 1:**
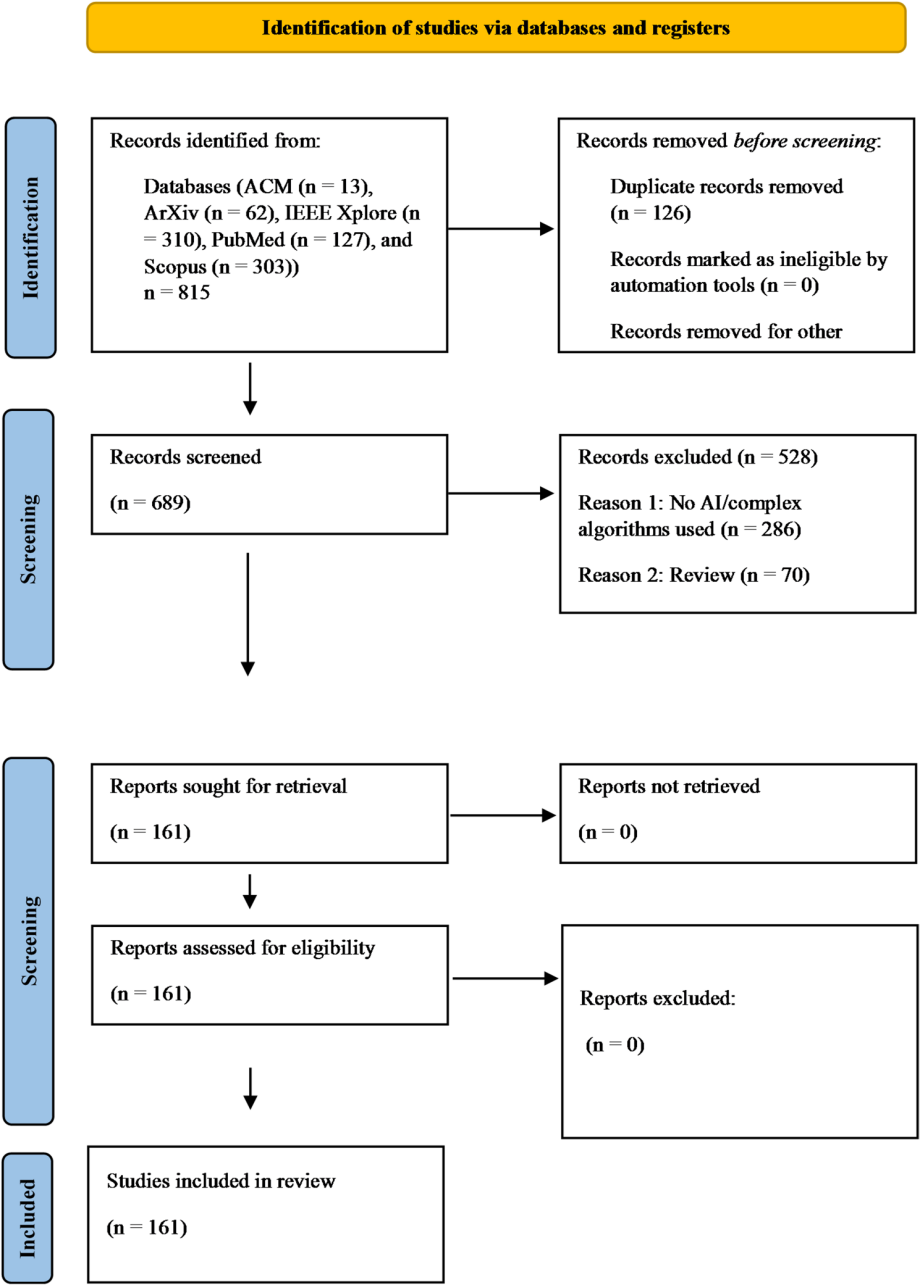
PRISMA [32] flow diagram: search and screening strategy

Our search yielded 815 eligible articles, which were imported into the open-source reference management software Zotero (version 6.0.30) for screening. The screening process followed the Preferred Reporting Items for Systematic Reviews and Meta-Analyses (PRISMA 2020) guidelines [32], as illustrated in Fig. 1. In the first step, titles and abstracts were assessed against predefined inclusion criteria (see Table 1). In the second step, full-text analysis was conducted on the 161 articles that passed the initial screening. We excluded articles published before 2010, non-English or non-German papers, non-original research (e.g., reviews, commentaries), non-peer-reviewed work, consumer product evaluations, and studies focused solely on technical feasibility or animal models. The 2010 cutoff ensured inclusion of research reflecting contemporary developments in AI and CL neurotechnology, while avoiding outdated approaches. In cases where algorithmic classification was ambiguous, we cross-checked and discussed the correct designation within our research team. The team consisted of a PI with a multidisciplinary background in philosophy, neuroscience, AI and biomedical ethics, a senior postdoc trained in both medicine and philosophy, and a PhD student in biomedical ethics with previous degrees in data science. Inclusion and exclusion decisions were carefully documented based on pre-determined criteria (see Table 1), with ambiguities resolved through team discussions. To ensure consistency and reliability, a random sample of 10% of the articles was independently verified. These measures reinforced the accuracy and reliability of the screening process.

**Table 1:** Inclusion and exclusion criteria overview

Inclusion criteria	Exclusion criteria
*Devices*: Neurostimulation devices, Neuromodulation devices, BCI, Neuroprostheses, Invasive and non-invasive devices, systems described to operate in a CL configuration	*Devices*: Consumer products
*Algorithm*: At least one AI or other complex algorithm	*Algorithm*: Simple algorithm, threshold-based algorithms, pure statistical algorithms
*Study subject*: Human subject, publicly available datasets of human-generated data (e.g., EEG datasets)	*Study subject*: Animal subject, Non-human technical feasibility research (like proof-of-concept)
*Article type*: Original articles, Case Reports, Clinical Trial, Randomized Controlled Trial, Conference Paper (only if presenting original work)	*Article type*: Reviews, Perspectives, Commentaries, Conceptual articles, Letters to the Editor, papers not publicly and freely available
*Language*: English and German	
*Published date*: 2010 until 12.01.2025	

A total of 161 articles met our inclusion criteria. Full texts were exported and analyzed using ATLAS.ti (version 24.1.1.30813) following a reflexive thematic analysis approach [33, 34]. The coding process was conducted iteratively and involved systematic categorization and refinement of codes through dynamic feedback and team discussions to ensure accuracy and consistency. In line with reflexive thematic analysis, we employed a combination of inductive and deductive strategies [35, 36]. Inductively, we applied open coding to identify recurring themes without imposing a pre-defined framework. This allowed the identification of relevant themes related to explainability and interpretability, even in cases where these terms were not explicitly mentioned but specific XAI methods were referenced. Deductively, our multi-professional team’s prior knowledge of XAI methods guided the coding process and informed the selection of material relevant to our research questions. All coding was jointly reviewed, and ambiguous cases were resolved collaboratively. In adherence to best practices for transparency and reproducibility, our review protocol was pre-registered on the Open Science Framework (OSF).

## Results

### Overview of included articles

**Fig. 2:**
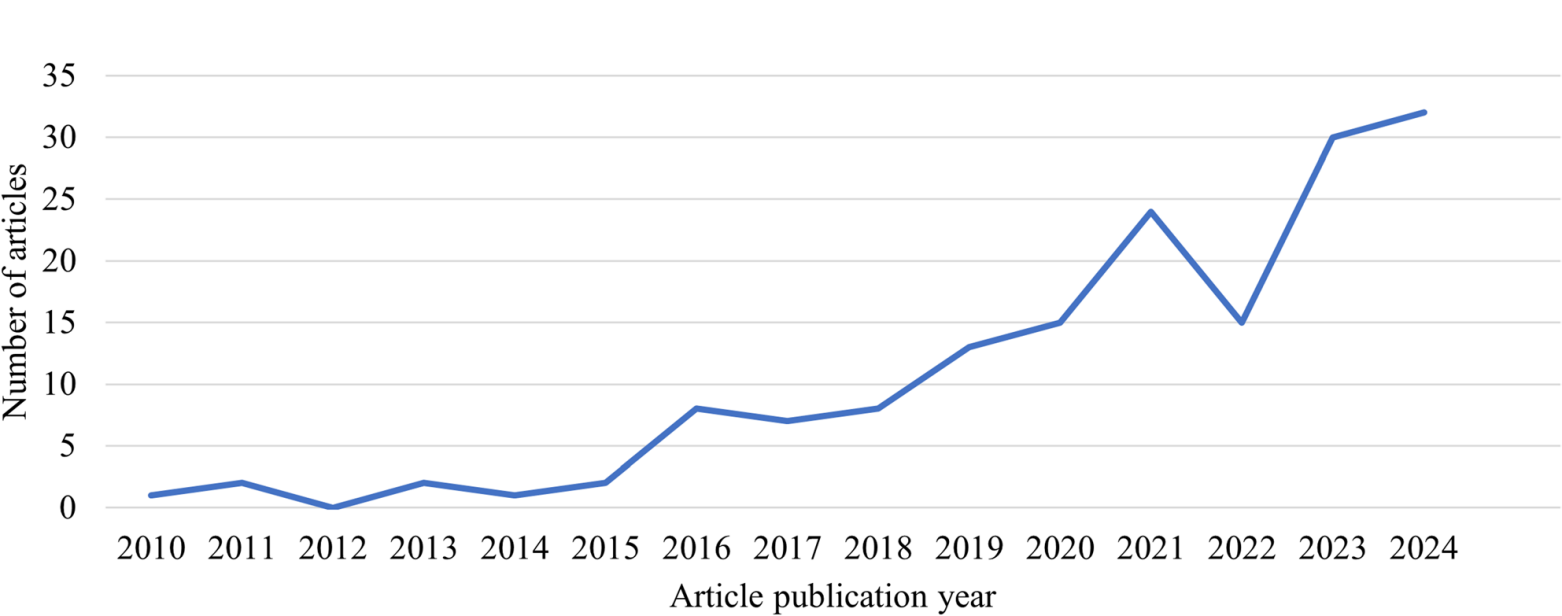
The number of articles (a total of 161) discussing medical CL neurotechnology and BCI increases over time. The search was conducted on Jan 12, 2025

Our findings indicate a substantial increase in the annual number of publications reporting on AI-enabled and other complex algorithm-based CL medical neurotechnology over the past decade. As shown in Fig. 2, the number of articles published between 2010 and 2024 steadily increased, peaking at 32 papers in 2024—the most recent complete year in our dataset.

The most common study populations included healthy individuals (*n* = 63), followed by patients with Parkinson’s disease (*n* = 28), epilepsy (*n* = 28), and stroke (*n* = 11). Among neurotechnologies, BCIs were the most frequently examined (*n* = 71), followed by DBS (*n* = 42), responsive neurostimulation (RNS) (*n* = 5), and transcranial magnetic stimulation (TMS) (*n* = 4). The majority of studies focused on the application of CL neurotechnology in neurological disorders such as Parkinson’s disease, epilepsy, or stroke rehabilitation (*n* = 139), whereas only relatively few studies investigated psychiatric uses, e.g. for depression or OCD (*n* = 16), or both (*n* = 6). Data types varied, with most studies using tabular data (*n* = 149), while a smaller subset utilized neuroimaging data (*n* = 15), including CT, MRI, and functional Magnetic Resonance Imaging (fMRI) scans. Sample sizes ranged widely, with many studies incorporating multiple datasets. Most articles (*n* = 92) reported sample sizes of 10 or more participants, while others focused on medium sample sizes of 5 to 9 participants (*n* = 33). A significant portion of studies (*n* = 53) reported on small sample sizes of fewer than 5 participants. Some articles addressed multiple conditions, neurotechnologies, data types, sample sizes, and algorithms. The algorithms most commonly employed across these neurotechnologies included Convolutional Neural Networks (CNNs) (*n* = 52), Support Vector Machines (SVMs) (*n* = 52), Dimensionality reduction techniques (*n* = 38), Tree-based models (*n* = 34), Linear Discriminant Analysis (LDA) (*n* = 30), and Recurrent Neural Networks (RNN) (*n* = 22).

Only 14 of the 161 reviewed articles (∼9%) reportedly employed XAI methods in conjunction with their underlying algorithms [37–50] (see Table 2). The reported XAI approaches included feature importance (*n* = 6), SHapley Additive exPlanations (SHAP) (*n* = 4), unspecified feature relevance (*n* = 2), Gradient-weighted Class Activation Mapping (GradCam) (*n* = 1), and one unspecified XAI method (*n* = 1). Across these studies, the use of XAI was inconsistently labeled. In cases lacking explicit terminology, classification as an XAI method was established through consensus among all authors, based on methodological characteristics.

Among the six studies leveraging feature importance plots or maps, the use case varied. One study analyzed key bandpower features from intracranial neural recordings in Parkinson’s disease patients [38], while another identified critical factors such as age and BMI for predicting complications in DBS surgery [43]. Another highlighted feature importance analysis as a tool for understanding brain mechanisms and translating insights into clinical DBS applications [46]. Another study emphasized the role of feature importance analysis in understanding brain mechanisms and translating this knowledge into clinical BCI applications [47]. Furthermore, another study looked at important features, specifically the top 5 features to predict clinical outcomes within Obsessive-compulsive disorder (OCD) patients [49]. In a related direction, one study generated a spatial feature importance map to identify the most relevant electrocorticography (ECoG) signals contributing to 3D hand translation [48]. Among the four studies employing SHAP, two used the technique to examine how individual input features influenced model predictions made by extreme gradient boosting (XGBoost) [42] and gradient-boosted trees [40]—both commonly applied in regression and classification tasks. Another study applied Shapley values to reduce dimensionality of input features when predicting tinnitus severity [37]. Similarly, SHAP analysis was used in a separate study to determine the most informative multimodal features for a regression model predicting obsessive-compulsive disorder (OCD) severity [50].

Two studies applied feature relevance techniques: one leveraged hand motor task data to classify ON versus OFF levodopa states in individuals with Parkinson’s disease [39], while the other employed Local Field Potentials (LFPs) for the same clinical classification [44]. One study employed GradCam, a widely used explainability method in computer vision, which utilizes gradient information from the final convolutional layer to highlight the most relevant feature among 64 input channels [45]. Although the MRMR (Minimum Redundancy Maximum Relevance) algorithm is not a canonical XAI method, it operates on a feature relevance logic [44]. Finally, one study reported using a generative causal explanation method to enhance biomarker discovery and to “[…] [explain] ‘black box’ machine learning models […]” [41].

**Table 2:** Overview of the fourteen XAI methods used in the reviewed articles

#	Type of XAI application	Authors and year	Neurotechnology	Trial Patient Group
1	Feature importance	Sendi et al., 2021 [46]	DBS	Depression
2	Feature importance	Farrokhi et al., 2020 [43]	DBS	Parkinson’s disease, Essential Tremor, Dystonia
3	Feature importance	Anjum et al., 2024 [38]	DBS	Parkinson’s disease
4	Feature importance	Gokulnath et al., 2024 [47]	BCI	No specific condition
5	Feature importance	Śliwowski et al., 2022 [48]	BCI	Hemi- or Tetraplegia
6	Feature importance	Olsen et al., 2020 [49]	DBS	OCD
7	SHAP	Fridgeirsson et al., 2023 [40]	DBS	OCD
8	SHAP	Hu and Beyeler, 2021 [42]	Neuroprosthetics	Retinitis pigmentosa
9	SHAP	Adcock et al., 2024 [37]	No specific system	Tinnitus
10	SHAP	Hinduja et al., 2024 [50]	DBS	OCD
11	Feature relevance	Castaño-Candamil et al., 2019 [39]	DBS	Parkinson’s disease
12	Minimal redundancy, maximal relevance feature selection algorithm (similar characteristics as feature importance/ feature relevance)	Sand, Rappel, et al., 2021 [44]	adaptive DBS (aDBS)	Parkinson’s disease
13	GradCam	Rajpura et al., 2024 [45]	BCI	NA
14	Generative causal explanations	Alagapan et al., 2023 [41]	DBS	Depression

### Thematic analysis

Our analysis revealed three main thematic families related to AI and other complex algorithms in medical CL neurotechnology.

(A) *Technical Constraints and Solutions*: Several studies examined hardware constraints that affect the implementation of XAI methods and proposed technological solutions to address these challenges.
(B) *Opacity and Explanation Quality*: Some studies addressed the challenge of algorithmic opacity and evaluated the quality of the explanations generated by AI systems. This theme captures the extent to which research efforts engage with the transparency of complex models and reflects a growing concern over the interpretability of decision-making processes in neurotechnology. The studies included under this theme not only identified the limitations posed by “black-box” models but also proposed or examined strategies to improve explanatory clarity, offering valuable insights into how AI transparency might be enhanced in clinical applications.
(C) *Impact on User Trust*: A smaller subset of studies explored the role of explainability in fostering trust in AI-enabled medical CL neurotechnology. While not a central focus in most studies, this emerging theme reflects a growing recognition of explainability’s importance in medical CL neurotechnology discourse.

#### Technical constraints and solutions

Studies on AI-enabled medical CL neurotechnology reported three key technical challenges: (1) the need for real-time processing/decoding in BCIs, (2) hardware constraints of neural implants, including limitations in memory, energy consumption, and computing power, and (3) potential solutions to address these constraints. Four studies identified real-time processing as a challenge, five highlighted hardware constraints, and four proposed cloud-based computing as a solution.

First, certain AI-enabled neurotechnologies, particularly BCIs, require real-time decoding of brain signals. Several studies emphasized that motor intent predictions must be processed instantaneously to benefit patients, such as those with tetraplegia [51]. For instance, the *Brain2Char* framework, which translates brain recordings into text, relies on real-time processing to ensure usability and enhance the user experience for patients [52]. One study highlighted that the processing time depends on the algorithm’s complexity and the hardware [53]. Another study underlined the fact that their discriminant analysis algorithm can be used for real-time BCIs [54].

Second, neural implants face various constraints in data storage, energy consumption, and computational capacity. Some studies emphasized the need for memory-efficient models for resource-limited applications [55]. For example, next-generation DBS devices are expected to feature expanded storage capabilities, enabling the deployment of more advanced algorithms to treat disorders such as Obsessive-Compulsive Disorder (OCD) [40]. To optimize energy consumption, one study suggested minimizing sensor usage in responsive DBS systems [56], while another highlighted how limiting EEG channels in seizure detection frameworks can significantly lower power demands in ambulatory care settings [57]. Algorithm complexity also plays a critical role in energy efficiency. Studies reported a clear trade-off between the performance of sophisticated seizure detection models and their impact on device battery life [55] with one study noting that elevated DBS stimulation parameters can accelerate battery depletion [58]. Furthermore, the computational limitations of implantable pulse generators (IPGs) necessitate the use of streamlined neural network architectures. These constraints require developers to balance model complexity with the need for real-time, on-device processing in neuromodulation applications, particularly for epilepsy patients [59].

Third, studies highlighted cloud-based computing as a viable solution to the computational constraints posed by large brain activity datasets. In seizure prediction, researchers emphasized that deep learning algorithms could be trained in the cloud using EEG and electrocorticography (EcoG) data to predict seizures and localize seizure foci [60, 61]. Electrocorticography enables direct cortical recordings of brain activity [62], which in turn can enhance predictive accuracy. Two studies specifically underscored the computational advantages of cloud-based real-time seizure prediction in BCIs for epilepsy patients [60, 61]. Ceteris paribus cloud-based approaches allow for greater computational power, which can overcome the limitations of local devices while enabling accessibility for multiple stakeholders [63]. Additionally, it facilitates transfer learning, allowing pre-trained models to be adapted to new patients with minimal retraining [63]. Overall, cloud-based approaches provide a scalable and flexible alternative to local hardware, enhancing the generalizability and accessibility of AI-enabled neurotechnologies for diverse clinical applications [63].

#### Opacity and explanation quality

Studies examined three interconnected dimensions: (a) the critical role of explainability in medical CL neurotechnology, (b) the dual challenge of algorithmic opacity and the inherent inscrutability of neurological and psychiatric conditions, and (c) proposed solutions like XAI techniques and interpretable models. Specifically, two studies differentiated among various types of explanations, eight studies emphasized the dual-layered opacity challenge, and nine studies proposed actionable strategies to enhance interpretability, including the use of XAI methods and linear modeling approaches.

The integration of AI and complex algorithms in medical CL neurotechnology necessitates a strong focus on explainability to meet the needs of diverse stakeholders. One study emphasized the importance of tailoring explanations to different audiences, as users require distinct types of information to effectively interpret and apply AI outputs [64]. Similarly, explainability in BCIs has been highlighted as essential for improving usability and mitigating frustration among users [45]. Our review identified two key dimensions of explainability in medical CL neurotechnology: (i) the opacity of the object being explained (the explanandum) and (ii) the value and quality of the explanation provided (the explanans). These themes were consistently observed across different types of medical neurotechnologies and their applications to both neurological and psychiatric conditions.

Eight studies highlighted a dual opacity challenge that complicates explainability in this field. The first aspect of this challenge involves the opacity of the algorithms themselves, with two studies observing that DL models often function as ‘black boxes’ [41, 42], producing accurate predictions with limited interpretability, thereby limiting their utility in clinical practice. The second aspect relates to the intrinsic complexity of the brain and neuropsychiatric conditions, adding another layer of uncertainty [57]. The brain itself is often regarded as a ‘black box’, with limited understanding of neural mechanisms and pathophysiological processes such as fronto-striatal activity in OCD [49, 65], or the therapeutic effects of DBS in Parkinson’s disease [66] and dystonia [67]. Third and more broadly, significant knowledge gaps remain in both the treatment and understanding of various neuropsychiatric conditions [68]. This knowledge gap may restrict AI models from processing meaningful input data, ultimately limiting their capacity to identify relevant patterns.

Overall, nine studies involved strategies to mitigate AI opacity and how to deal with it in general in medical CL neurotechnology. Studies diverged with regard to the strategies that can be employed to reduce algorithmic opacity. While the first group discussed and implemented specific XAI techniques, the second group favored inherently interpretable models.

The first group emphasized the need of identifying contributing features and demonstrated how XAI methods can enhance explainability by visualizing the predictions made by AI algorithms [40, 42]. For example, SHAP values were used to help clinicians trace the ' inner-workings’ of models and understand how conclusions are derived [37]. Additionally, some studies advocated for incorporating reliable biomarkers as model inputs, such as High Order Spectrum (HOS)-related biomarkers, which have been shown to enhance sensitivity and explainability of AI-powered wearables for detecting Parkinson’s disease tremor properties [69]. Additionally, one study stressed the need to tailor explanations to stakeholders to ensure relevance and usability [45].

In contrast, a second group advocated for inherently interpretable models for classification tasks in medical CL neurotechnology, highlighting resource-efficient approaches like oblique trees (ResOT) [55] or linear models [70]. These preferences were reflected in practice, as seen in the use of LDA in Parkinson’s disease studies [39]. Falling between these two approaches are studies that emphasize balancing interpretability and performance as key criteria for model selection [71]. For instance, the choice between LDA and SVM often depends on both the ease of understanding the model and its predictive accuracy. While linear models are considered to be more interpretable, more complex AI models such as SVM typically deliver superior performance, which can be crucial in applications such as AI-enabled BCIs for stroke rehabilitation [70]. Despite these efforts to improve interpretability, opaque models like DL remain highly relevant and are still employed in scenarios where performance relies on the sophistication of the algorithm, such as in using DBS systems for the treatment of OCD [40]. These findings underscore the importance of balancing explainability and performance needs while adapting strategies to the specific demands of medical CL neurotechnology.

#### Impact on user trust

The included studies explored the influence of model complexity on the usability and acceptance of AI systems, while also emphasizing the need for further research to address persistent challenges in the field. Several studies underscored the importance of explainability in fostering trust in AI-driven decision-making, and others called for deeper investigation into methods that could enhance the reliability and interpretability of these technologies. Four studies report that concerns about the explainability of AI-enabled medical CL neurotechnology extend beyond technical aspects and model interpretability into broader issues of user trust and clinical acceptance. Some noted that clinicians may distrust complex AI models due to their opacity [43], emphasizing the need for *algorithmic transparency* in medical applications [39]. Others argued that enhancing feature extraction interpretability could improve clinicians’ confidence in DL models compared to generalized DL [72]. One study labeled its approach as a trustworthy epilepsy prediction technique, demonstrating that Deep Belief Networks (DBNs) reliably predict seizure likelihood [73]. DBNs, known for learning hierarchical representations, outperformed other feature selection methods in explanatory power, increasing their clinical credibility [73].

Four studies underscored the need for further research to improve explainability, model accuracy, and interpretability while ensuring trustworthy AI solutions [40, 43, 47, 74]. One study specifically called for enhancing the explainability of Extreme Gradient Boosting Machines (XGBMs), a widely used gradient boosting algorithm which builds a series of decision trees sequentially [43]. Another highlighted the need for explainability techniques that enhance model visualization and interpretation, providing clearer insights into symptom identification and disease understanding [40]. Moreover, another study called for future research to focus on developing user-friendly and transparent AI solutions, particularly in epilepsy management, to ensure both clinical effectiveness and trust [74]. Finally, one study emphasized that ethical considerations like data privacy and informed consent should be prioritized in future research [47] (see Table 3).

**Table 3:** Characteristics of articles included in the scoping review

#	Reference (Name, Year)	Country	Sample Size	Data type	Condition	Neurotechnology	Algorithm	XAI considerations
1	A. T. Nguyen et al., 2021 [75]	USA	1	Neural data	Transradial amputee	Neuroprosthesis	GRU	None
2	Adcock et al., 2024 [37]	Ireland, UK, USA	432	Tabular data with descriptive features	Tinnitus	No specific system	CNN, Decision tree, Linear regression, RF, SVM	Apply XAI method (although not calling it XAI)
3	Adithya et al., 2024 [76]	India	5	EEG	Healthy individuals	BCI	CNN-LSTM, PCA	None
4	Afzal et al., 2024 [77]	Switzerland, USA	24	EEG	Epilepsy disorder	No specific system	CNN-LSTM, DCRNN, GRU, LSTM, REST algorithm, RNN, Transformer	None
5	Aiello et al., 2023 [78]	Switzer-land	3	Perceptual threshold	Trans-femoral amputee	The tibial branch of the sciatic nerve stimulation	Gaussian process-based Bayesian optimization method, K-Means clustering	Use XAI nomenclature like robustness and ‘black box’
6	Ajra et al., 2024 [79]	France	12, 29, 25	EEG	Healthy individuals	BCI	CNN	None
7	Akbar et al., 2020 [80]	Spain, USA,	1	Motor evoked potential	Healthy individual	TMS system	CNN	None
8	Alagapan et al., 2023 [41]	USA	6	Electrophysiolo-gical data	Depression	DBS	LR, RF, Neural network classifier	Apply XAI method
9	An et al., 2024 [81]	Australia, China	9, 5	EEG	Healthy individuals	BCI	CNN, CSP, Diffusion model, Reinforcement Learning	None
10	Ang and Guan, 2017 [82]	Singapore	34	EEG	Stroke	BCI	CSP, FBCSP	None
11	Anjum et al., 2024 [38]	UK, USA	4, 1	ECoG	Parkinson’s disease, Cervical dystonia	DBS	SVM	Apply XAI method (although not calling it XAI)
12	Askari et al., 2024 [83]	USA	3	Magnetencepha-lography scan, EEG	Epilepsy disorder	RNS system	ICA	None
13	Aslan and Yilmaz, 2024 [84]	Kuwait, Turkey	30	EEG	Healthy individuals	BCI	AdaBoost, Bagging, CNN, Decision Tree, DNN, ICA, Kernel, KNN, NB, RF, SVM,	None
14	Astrand et al., 2021 [85]	Sweden	2	EEG	Stroke	MI-BCI	SVM	None
15	Avvaru et al., 2021 [86]	USA	10	Local field potential (LFP) recordings	Epilepsy disorder	No specific system	SVM	None
16	Bangroo and Tahzeen, 2023 [74]	India, USA	1, 5	EEG	Epilepsy disorder	RNS system	LR, RF, SVM	None
17	Bao et al., 2022 [87]	China	27, 2	EEG	Healthy individuals, NA	MI-BCI	Canonical correlation analysis, SVM, DNN	Use XAI nomenclature (interpretability) and apply interpretable hybrid DL method
18	Behboodi et al., 2024 [88]	USA	8, 1	EEG	Healthy individuals, Cerebral palsy patient	BCI, Neuromuscular electric stimulator (NMES)	LSTM, MLP	None
19	Bhargavi et al., 2019 [89]	India	15	EEG	Healthy individuals	Neurosky midwave headset	DNN, RNN, SVM	None
20	Boutet et al., 2021 [90]	Canada, India, USA	67	fMRI	Parkinson’s disease	DBS	LDA	None
21	Bouton et al., 2016 [91]	USA	1	fMRI	Quadriplegia	Neuroprosthesis	SVM	None
22	Castaño-Candamil et al., 2019 [39]	Germany	4	EEG	Parkinson’s disease	DBS	LDA	Apply XAI method
23	Cernera et al., 2021 [56]	USA	10	EMG	Essential Tremor	DBS	SVM	None
24	Cherukuvada and Kayalvizhi, 2023 [73]	India	23	EEG	Epilepsy disorder	No specific system	CNN, DBN, DL-based approach, LR, LSTM-DBN, SVM	Use XAI nomenclature. In this case trust
25	Chintamani et al., 2021 [92]	India	9	EEG	Healthy individuals	BCI	CSP, Probability weight deep neural network, Wavelet packet decomposition	None
26	Cho et al., 2024 [93]	Japan, Taiwan	NA	Synaptic current	Parkinson’s disease, Healthy individuals	DBS	Reinforcement Learning	None
27	Cohen et al., 2023 [94]	Denmark, Israel	16	Neural activity	Parkinson’s disease	DBS	Diffusion maps, K-Means clustering, Unsupervised State Variables approximation (USVA), Hidden Markov model	None
28	D. Li et al., 2022 [95]	China	12	EEG	Healthy individuals	MI-BCI	CNN	None
29	Desbois et al., 2024 [96]	France	2	EEG	Healthy individuals	BCI	LDA	Use XAI nomenclature. In this case interpretability
30	Elgharabawy and Wahed, 2016 [97]	Egypt	3	ECoG	Epilepsy disorder	BCI	Gram-Schmidt feature selection technique, Linear regression, SVM	None
31	Engelhardt et al., 2021 [98]	France	43	MR image	Essential Tremor	DBS	Support vector regression, Reproducing Kernel Hilbert space (RKHS): kernel ridge regression, DNN	None
32	Even-Cheng et al., 2018 [71]	USA	1, 1	LFP recording	Amyotrophic lateral sclerosis, Spinal cord injury	iBCI	Hidden Markov model state classifier, LDA, PCA	Use XAI nomenclature. In this case interpretability
33	Fan et al., 2024 [99]	USA	1	Threshold-crossing neural features	Spinal cord injury (SCI)	iBCI	GRU, LLM	None
34	Farrokhi et al., 2020 [43]	Australia, Canada, USA	501	Tabular data with descriptive features	Parkinson’s disease, Essential Tremor, Dystonia, Other indications	DBS	XGBM, LR, Synthetic Minority Oversampling Technique	Apply XAI method (although not calling it XAI)
35	Flavin et al., 2022 [100]	USA	109	EEG	Healthy individuals	BCI	CNN, KNN, LDA, LR, MLP, Naive Bayes Classifier, RF, SVM, XGBM	None
36	Fløtaker et al., 2023 [101]	Norway	31	EEG	Healthy individuals	BCI	CNN, Graph Convolutional Neural Network	None
37	Fonseca et al., 2018 [102]	Brazil, France	8	Accelerometer and gyroscope data	Hemi- or Tetraplegia	Robotic hand	PCA	None
38	Franke et al., 2024 [103]	Republic of Korea, USA	6, 10, 4	Electric Field	Healthy individuals	TMS system	CNN	None
39	Fridgeirsson et al., 2023 [40]	The Nether-lands, UK, USA	11	LFP recording	OCD	DBS	Gradient-boosted tree, InceptionTime	Apply XAI method
40	Gabardi et al., 2023 [104]	Italy	52	EEG, EMG, Electrooculography	NA	No specific system	CNN, Fully convolutional neural network, RNN	None
41	Gabriel et al., 2019 [105]	USA, Germany, Grenada	3	ECoG, IEEG, Electroencephalo-graphy (sEEG)	Epilepsy disorder	No specific system	LDA, MLP, SVM	None
42	Galván et al., 2024 [106]	Argentina	54,2	EEG	NA	BCI	CNN, CSP, Generative Adversarial Network	None
43	Gao et al., 2021 [107]	China	18	Event-related potentials	Healthy individuals	BCI	LDA	None
44	Ghodratito-ostani et al., 2022 [108]	Brazil, Iran, USA	6	EEG, fMRI	Tinnitus	Transcranial Electrical Stimulation (tES)	Adaptive Seamless Bayesian method	None
45	Girdher et al., 2020 [109]	India	15	EEG	Healthy individuals	BCI	DBN, DNN, ICA, LDA, LR, Naive Bayes Classifier, PCA, RF, Restricted Boltzmann Machine, SVM	None
46	Giri et al., 2024 [110]	India	9	EEG	Healthy individuals	BCI	LR, RF, SVM	None
47	Gokulnath et al., 2024 [47]	India	20	EEG	Healthy individuals	BCI	CNN, LSTM, RNN	Apply XAI method (although not calling it XAI)
48	Gong et al., 2023 [111]	China	12	LFP recording	Parkinson’s disease	DBS	CNN, RF, SVM	None
49	Goudman et al., 2023 [112]	Belgium, France	250	Spinal cord related data (pain intensity scores, functional disability, medication use etc.)	Chronic pain	SCS system	LDA, Quadratic discriminant analysis	None
50	Gruenwald et al., 2019 [54]	Austria, Japan, Russia	6	ECoG	Epilepsy disorder	BCI	CSP, LDA, PCA	None
51	Harati et al., 2018 [113]	USA	13	Voice of patient	Depression	DBS	LSTM, SVM, KNN, Hidden Markov Model with Gaussian Mixture Emissions, RNN	None
52	Haxel et al., 2025 [114]	Germany	50	EEG, EMG	Healthy individuals	TMS system	LR, RF, SVM	Use XAI nomenclature. In this case interpretability
53	Hinduja et al., 2024 [50]	USA, The Netherlands	6	Multimodal	OCD	DBS	RF	Apply XAI method
54	Höhne et al., 2014 [115]	Germany, Korea	4	EEG	Hemi- or Tetraplegia	BCI	CSP, LDA	None
55	Hooper et al., 2017 [116]	USA	4	IEEG	Epilepsy disorder	New neurostimulation system (intracranial electrodes and chip e.g., behind the ear)	SVM	None
56	Hosman et al., 2023 [117]	USA	1	Intracortical neural activity	Hemi- or Tetraplegia	iBCI	LSTM	None
57	Hosseini et al., 2016 [60]	Iran, USA	2	iEEG	Epilepsy disorder	BCI	PCA, Stacked autoencoder	None
58	Hosseini et al., 2017 [61]	Iran, USA	9, 2	EcoG, EcoG	Epilepsy disorder	BCI	Autoencoder, CNN, Differential Search Algorithm, ICA, MLP, PCA, RF, SVM	None
59	Houston et al., 2015 [66]	USA	1	LFP recording	Parkinson’s disease	DBS	LR	None
60	Hu and Beyeler, 2021 [42]	USA	12	Tabular data with descriptive features	Retinitis pigmentosa	Argus II retinal prosthesis system	LR, SVM, XGBM	Apply XAI method
61	Huang and Lin, 2023 [118]	Taiwan	10	EEG	Healthy individuals	Affective BCI	Quadratic regression, SVM	None
62	Hussain Shah et al., 2021 [63]	Saudi Arabia, USA	109	EEG	Healthy individuals	BCI	CNN	None
63	Irimia et al., 2017 [119]	Austria, Romania	2	EEG	Stroke	Functional electrical stimulation (FES)	CSP, LDA	None
64	Iyer and Somappa, 2024 [58]	India	14	EEG	Epilepsy disorder	No specific system	GRU	None
65	Jia et al., 2023 [120]	China	9	EEG	Healthy individuals	MI-BCI	CNN, LightGBM	Use XAI nomenclature. In this case interpretability
66	Kamble, Ghare, and Kumar, 2023 [121]	India	15	EEG	Healthy individuals	Automatic Imagined speech recognition system	CNN	None
67	Kamble, Ghare, Kumar, et al., 2023 [122]	India	15	EEG	Healthy individuals	Automatic imagined speech recognition (AISR) system	CNN	None
68	Kang et al., 2023 [123]	Czech Republic, China, Hong Kong, UK	52, 48	EEG	Healthy individuals, Depression	BCI	Residual neural network	None
69	Kavoosi et al., 2022 [59]	UK	2	LFP recording	Epilepsy disorder	No specific system	CNN, Filter-based spectral biomarker detector, MLP	None
70	Khan et al., 2023 [124]	Pakistan, USA	15	EEG	Healthy individuals	BCI	CNN	None
71	Khattar and Kaur, 2024 [125]	India	NA	EEG	NA	No specific system	CNN, ICA, KNN, LSTM, RF, SVM	None
72	Kiakojouri et al., 2020 [126]	Iran	1	EEG	Healthy individual	TMS system	MLP	None
73	Kim et al., 2020) [127]	USA	60	MR image	Healthy individuals	No specific system	CNN	None
74	Klempíř et al., 2018 [67]	Czech Republic	13	MER signal	Dystonia	DBS	LR, Naive Bayes Classifier, SVM	None
75	Koch et al., 2019 [128]	Germany, The Nether-lands	40	EEG	Parkinson’s disease	DBS	RF	None
76	Kumar Saidala et al., 2024 [129]	India	NA	EEG	NA	BCI	DBN, Residual Neural Network	None
77	L. Wang et al., 2023) [130]	China	9, 14	EEG	Healthy individuals	BCI	CNN	None
78	Leguia et al., 2021 [131]	Switzer-land, USA	222	Continuous EEG	Epilepsy disorder	RNS system	K-Means clustering, Nonnegative matrix factorization	None
79	LeMoyne et al., 2020 [132]	USA	1	Acceleration magnitude signal	Parkinson’s disease	DBS	MLP	None
80	Li and Nie et al., 2024 [53]	China	1	LFP	Parkinson’s disease	DBS	ML algorithm but not specified	None
81	Li et al., 2024 [133]	China	2	EEG	NA	BCI	CNN	None
82	Liao et al., 2022 [134]	USA	3	EEG	Healthy individuals	No specific system	RF, Mixture Independent Component Analysis	None
83	Liu and Dawant, 2016 [135]	USA	100	MR image	Parkinson’s disease	DBS	Regression Forest	None
84	Liu et al., 2016 [136]	China, USA	5, 5, 9	EEG	Stroke	BCI	CSP, Common spatial-spectral boosting pattern, SVM	None
85	Luckett et al., 2019 [57]	USA	40	Electroencephalography (sEEG)	Epilepsy disorder	No specific system	CNN	None
86	Luu et al., 2017 [137]	USA	12	EEG	Healthy individuals	BCI	K-Means clustering	None
87	Majid Mehmood et al., 2017 [138]	China, South Korea	21	EEG	Healthy individuals	Human-computer interaction	Ensemble methods, DNN, KNN, LDA, Naive Bayes Classifier, RF, SVM	None
88	Manoj et al., 2023 [139]	India	756	Voice of patient	Parkinson’s disease	No specific system	LR, PCA, KNN, SVM, Gradient Boosting Classifier algorithm, RF, Ensemble methods	None
89	Maurer et al., 2016 [140]	USA	1	EEG	Parkinson’s disease	DBS	Matching pursuit decomposition, GMM	None
90	Mello et al., 2022 [141]	USA	122	EEG	Alcoholism	BCI	CNN, DNN, Gradient boosting, LDA, LR, RF, Riemannian minimum distance to mean	None
91	Meng et al., 2022 [142]	Australia, South Korea	5	EEG	Epilepsy disorder	BCI	LDA	None
92	Meng et al., 2023 [143]	Australia, Republic of Korea	10, 10	EcoG, sEEG	Epilepsy disorder	Speech neuroprostheses	LDA, Multivariate temporal response function, Single pass spectrogram inversion, Short-Term fourier transform	None
93	Metzger et al., 2023 [144]	USA	1	ECoG	Stroke	BCI	Beam-search algorithm, Linear regression, RNN	None
94	Mohanty et al., 2018 [70]	USA	20	Neuroimaging data	Stroke	BCI	Support vector regression	Use XAI nomenclature. In this case interpretability
95	Mou et al., 2024 [145]	China	3, 9	EEG	NA	BCI	CNN, FBCSP, Hierarchical window attention network	None
96	Mughal et al., 2021 [146]	Pakistan	26	FNIRS	Healthy individuals	BCI	CNN-LSTM	None
97	Naros et al., 2016 [147]	Germany	32	EEG	Healthy individuals	Brain-robot interface, Transcranial direct current stimulation (tDCS)	Reinforcement Learning	None
98	Navarro-Sune et al., 2016 [148]	Australia, France, UK	9	EEG	Healthy individuals	Brain-ventilator interface	CSP, K-Means clustering, LDA, SVM	None
99	Neudorfer et al., 2021 [149]	Canada, Germany, USA	58	MR image	Alzheimer’s disease	DBS	LR, Multivariate regression analysis, KNN	None
100	O’Leary et al., (2017) [150]	Canada	300	iEEG	Epilepsy disorder	No specific system	SVM	None
101	O’Leary et al., 2018 [151]	Canada, USA	30	iEEG	Epilepsy disorder	RNS system	Autoencoder, PCA, SVM	None
102	Oehrn et al., 2024 [152]	USA	4	STN spectral gamma power	Parkinson’s disease	DBS	LDA, Linear regression	None
103	Olsen et al., 2020 [49]	USA	1	LFP recording	OCD	DBS	Linear regression, RF	Apply XAI method (although not calling it XAI)
104	Oxley et al., 2021 [153]	Australia, USA, UK	2	ECoG	Amyotrophic lateral sclerosis (ALS)	BCI	SVM	None
105	Ozturk et al., 2020 [154]	USA	10	LFP recording, Microelectrode single unit activity (SUA) recordings	Parkinson’s disease	DBS	LDA	None
106	P. Nguyen et al., 2013 [155]	Australia	40	EEG	Healthy individuals	No specific system	SVM	None
107	Peralta et al., 2020 [156]	Canada, France	57	Microelectrode recording (MER) signal	Parkinson’s disease	DBS	CNN	None
108	Petersen et al., 2018 [157]	Denmark	3	EEG	Healthy individuals	BCI	FBCSP, MLP, Separable Common Spatio-Spectral Pattern algorithm	None
109	Provenza et al., 2021 [65]	The Nether-lands, USA	5	Intracranial electrophysiolo-gical recording	OCD	DBS	Automated Facial Affect Recognition (AFAR) algorithm (based on CNN)	None
110	Qiu et al., 2024 [158]	Canada, USA	39	fMRI	Parkinson’s disease	DBS	Autoencoder	None
111	Rajpura, Cecotti, and Meena, 2024 [45]	USA, India	109	EEG	Healthy individuals	BCI	CNN, Riemannian minimum distance to mean	Apply XAI method
112	Ranjani and Supraja, 2021 [159]	India	8, 12, 18	EEG	Autism, Epilepsy disorder, Healthy individuals	BCI	ICA, CNN	None
113	Rathore et al., 2019 [160]	Qatar, USA	16	Rest tremor velocity (RTV)	Parkinson’s disease	DBS	LSTM	None
114	Roediger et al., 2023 [161]	Germany	35	CT image, MR image	Parkinson’s disease	DBS	StimFit algorithm	None
115	Saif-Ur-Rehman et al., 2019 [162]	Germany, USA	8	Neural data (single unit activity)	Epilepsy disorder	No specific system, BCI	CNN, DNN, GMM, PCA	None
116	Sand, Arkadir, et al., 2021 [163]	Israel, USA	17	EEG	Parkinson’s disease	DBS	SVM	None
117	Sand, Rappel, et al., 2021 [44]	China, Israel, USA	4	Neuronal signal data	Parkinson’s disease	DBS	DNN, Linear regression, SVM	Apply XAI method
118	Sang et al., 2024 [164]	China	13	CT images	NA	DBS	Skeletonization algorithm	None
119	Sarikhani et al., 2019 [165]	USA	10	DBS Stimulation amplitude, stimulation contact, tremor rating	Parkinson’s disease	DBS	Gaussian Process (GP) regression model, Multinomial LR	None
120	Scangos et al., 2021 [68]	USA	1	IEEG	Major depressive disorder	DBS	Linear regression, LR, K-Means clustering	None
121	Schreuder et al., 2023 [166]	Germany, Italy, UK	1	MR image	Stroke	BCI	LDA	None
122	Sendi et al., 2021 [46]	USA	8	Stimulation amplitude (DBS), Electrophysio-logical data	Depression	DBS	LR	Apply XAI method (although not calling it XAI)
123	Sharma and Somappa, 2024 [167]	India	10	EEG	Epilepsy disorder	RNS system	Neural tree classifier (Hybrid DT and NN), SVM	None
124	Sheykhivand et al., 2020 [168]	Iran, Malaysia	16	EEG	Healthy individuals	BCI	CNN-LSTM, Deep Boltzmann Machine, MLP	None
125	Shin et al., 2022 [169]	USA, Switzer-land	1, 24	IEEG	Parkinson’s disease, Epilepsy disorder	BMI	NeuralTree classifier	None
126	Shoji et al., 2021 [170]	Japan	19	EEG	Epilepsy disorder	No specific system	CNN, SVM	None
127	Simeral et al., 2011 [171]	USA	1	LFP recording	Stroke	BCI	LDA, Linear regression	None
128	Singh et al., 2023 [172]	India	1	EEG	Healthy individual	BCI	LDA	None
129	Śliwowski et al., 2022 [51]	France	1	ECoG	Hemi- or Tetraplegia	BCI	CNN, CNN-LSTM, MLP, Multilinear model	Apply XAI method (although not calling it XAI)
130	Śliwowski et al., 2023 [173]	France	1	ECoG	Hemi- or Tetraplegia	BCI	CNN-LSTM, MLP, Multilinear model, SVM, Uniform Manifold Approximation and Projection	None
131	Sonnet et al., 2020 [174]	USA	3, 2	Line segments, average pressure	Essential Tremor, Healthy individuals	DBS	Gradient boosting classifier, LDA	None
132	Stachaczyk et al., 2019 [175]	UK	1	EMG	Healthy individual	Neuroprosthesis	LDA	None
133	Stachaczyk et al., 2020 [176]	UK, USA	5	EMG	Healthy individuals	No specific system	LDA	None
134	Stuart et al., 2019 [177]	USA	16	EEG	Dystonia, Parkinson’s disease, Tourette Syndrome	DBS	CSP, Feature clustering, Gaussian radial basis function kernel (GRBF), Gradient-boosted tree, KNN, ICA, LR, PCA, RF, SVM	None
135	Sun et al., 2020 [52]	USA	4	ECoG	Healthy individuals	BCI	CNN	None
136	Suri et al., 2023 [178]	India	5	EEG	Healthy individuals	MI-BCI	KNN, SVM, Ensemble methods, Decision Tree	None
137	T.D. Pham, 2023 [179]	Saudi Arabia	25	EEG	Healthy individuals	MI-BCI	CNN, CSP, FBCSP, LSTM, SVM	None
138	Tan et al., 2024 [180]	China	9	EEG	Healthy individuals	BCI	CNN	None
139	Tao et al., 2023 [181]	People’s Republic of China	13	EEG	Healthy individuals	MI-BCI	CSP, Fisher’s discriminant analysis, LDA, SVM	None
140	Velasco et al., 2022 [182]	USA	9	LFP recording	Parkinson’s disease	DBS	Adaptive time segmentation (Similar to random forest/decision tree)	None
141	Vlek et al., 2011 [183]	The Nether-lands	10	EEG	Healthy individuals	No specific system	LR, PCA	None
142	Wang et al., 2022 [184]	China, USA	13	EEG	Patient during anesthesia	No specific system	CNN, LSTM, RF, SVM	Use XAI nomenclature. In this case interpretability
143	Wang et al., 2023 [185]	China	8	EEG	Healthy individuals	No specific system	CNN	None
144	Wang et al., 2024 [186]	China	10	EEG	Healthy individuals	BCI	CSP, SVM	None
145	Wei et al., 2010 [187]	China	3	ECoG	Epilepsy disorder	BCI	Genetic algorithm-based wrapper method, CSP, PCA, Fisher discriminant analysis	None
146	Weng et al., 2024 [188]	China	157, 159	sMRI	Healthy individuals	DBS system	CNN	None
147	Werneburg et al., 2023 [189]	USA	381	Tabular data with descriptive features	Overactive Bladder	Sacral neuromodulation (SNM)	DNN	None
148	Wu et al., 2022 [190]	Denmark	9, 109	EEG	Healthy individuals	BCI	Blind source separation technique, ICA, K-Means clustering, Linear regression	None
149	Wu et al., 2024 [191]	China	500, 2, 10	EEG, EMG	Epilepsy disorder, Healthy individuals, Healthy individuals	No specific system	CNN, Fourier inversion prediction, Transformer	None
150	X. Jiang et al., 2019 [192]	China, UK	29	EEG, fNIRS	Healthy individuals	BCI	SVM, CSP, LDA, Hidden Markov Model	None
151	X. Zhang et al., 2019 [193]	Australia, USA	20, 8, 5	EEG	Epilepsy disorder	BCI	AB, CNN, GRU, KNN, LDA, LSTM, Reinforcement Learning, RF, SVM	None
152	Y. Li et al., 2021 [194]	China	9, 14	EEG	Healthy individuals	BCI	CNN	None
153	Yu et al., 2015 [195]	China	3, 16	EEG	Healthy individuals	BCI	Bayesian linear discriminant analysis, Fisher Criterion Ratio, Lasso regularized least square, Recursive channel elimination	None
154	Z. Jiang et al., 2021 [72]	USA	12	Face images of patients	Depression	DBS	CNN, Linear regression, LR, SVM, Gradient-boosted decision tree, LSTM	Use XAI nomenclature. In this case trust and interpretability
155	Z. Zhang et al., 2018 [196]	Singapore	13	EEG	Stroke	MI-BCI	CSP, FBCSP	None
156	Zhang et al., 2021 [197]	China	1	EEG	Healthy individual	BCI	CSP, KNN, NB, RF, SVM	Use XAI nomenclature. In this case interpretability
157	Zhang et al., 2022 [198]	China, Singapore	14, 19	EEG	Stroke	BCI	CNN, FBCSP	None
158	Zhang, Ang et al., 2022 [199]	China, Singapore	33, 9, 14	EEG	Stroke, Healthy individuals, Healthy individuals	BCI	CNN, FBCSP, KNN	None
159	Zheng et al., 2024 [200]	China	25, 66	EEG	Healthy individuals, Cochlear Implant patients	Neural prosthesis	SVM	None
160	Zhu, Farivar, and Shoaran, 2020 [55]	Switzer-land, USA	10, 12, 9	iEEG, ECoG	Epilepsy disorder, Parkinson’s disease, Healthy individuals	DBS, BMI	Decision tree (oblique), LightGBM, PEGB, quantized PEGB	Use XAI nomenclature. In this case interpretability
161	Ziogas et al., 2023 [69]	Greece, United Arab Emirates	16	Index finger velocity recordings	Parkinson’s disease	DBS	LR, K-Means clustering, KNN, PCA, SVM	Use XAI nomenclature. In this case explainability

Abbreviations of Data types:* fNIRS* Functional near-infrared spectroscopy,* iEEG *Intracranial electroencephalography, *LFP* Local field potential, *MER *Microelectrode recording. Abbreviations of algorithms: *CSP *Common Spatial Pattern, *DNN* Deep Neural Network, *FBCSP* Filter bank common spatial pattern, *GMM *Gaussian Mixture Model, *GRU* Gated Recurrent Unit, *ICA *Independent component analysis, *LightGBM *Light Gradient-Boosting Machine,* LR* Logistic Regression, *MLP* Multilayer Perceptron, *PCA* Principal Component Analysis, *PEGB* Power-efficient gradient boosting, *RF *Random Forest. Note: Filtering, transformation (e.g., Fourier-Transformation), and artifact removal methods not central to the article are not included in the algorithm section

## Discussion

Despite the widespread recognition of explainability as an essential requirement for AI in medical technology, our scoping review reveals that the prevalence of XAI approaches to AI-enabled medical CL neurotechnology is remarkably low (∼9%). This discrepancy is striking, given the well-documented challenges posed by algorithmic opacity, the critical role of high-quality explanations, and the established benefits of explainability in improving both the functionality and ethical integrity of neurotechnological devices.

Explainability is not just a technical concern but a central ethical consideration in healthcare, particularly in contexts where clinicians and patients rely on AI-supported decisions. It supports key principles such as informed consent, fairness, and accountability by exposing biases in decision-making processes and ensuring transparency in patient care [10, 201–204]. Enhancing transparency and trust in AI systems is also considered crucial for their wider clinical adoption [205]. Without at least a minimal level of explainability, AI and complex algorithm decision-making processes remain opaque, which can undermine the trust of both clinicians and patients in these systems [201]. As medical neurotechnologies continue to evolve, incorporating XAI emerges as a moral and medical imperative. By making these complex systems more understandable and accountable, XAI methods can lead to, when not over-relied on, enhanced patient outcomes and strengthen clinical practices [206].

However, the current lack of XAI implementation identified in this review highlights a significant opportunity for future research and development in this field, where explainability will be a critical factor in ensuring both the efficacy and ethical integrity of AI-enabled and complex algorithm-based neurotechnological solutions. In the realm of explainability in the context of medical CL neurotechnology, it remains crucial to further evaluate what specific types of explanations should be provided by developers for what explanation goal [207, 208]. A central question raised by our findings is why existing work on medical CL neurotechnology has not implemented XAI methods more extensively, despite widespread acknowledgment of their importance. Three primary factors emerge from our findings: hardware limitations, software performance trade-offs, and stakeholder concerns.

At the hardware level, the constraints of current devices inhibit the integration of XAI into neurotechnological systems. Energy consumption, battery limitations, and computational capacity present significant barriers. Advancements such as rechargeable batteries in neurostimulation devices could be critical for supporting the integration of XAI and other complex algorithms into implantable pulse generators. In parallel, cloud-based solutions can alleviate local hardware constraints by offloading computational tasks, thus allowing for the implementation of advanced algorithms such as anomaly detection and pattern recognition, which are essential for both improving patient outcomes, enhancing technical explainability, and improving both data analysis and model interpretation [60]. However, the increased use of cloud-based solutions in combination with the growing volume of brain data raises privacy and security concerns surrounding data storage and processing. Among the current hardware constraints, concerns surrounding brain data privacy and general data security are likely to persist as critical ethical challenges. In contrast, limitations related to computational power and battery life may be mitigated through technological advancements and emerging paradigms such as neuromorphic AI [209]. However, to what extent any of the current constraints resolve remains speculative.

However, the need to balance technological innovation with privacy and security considerations is paramount. Ensuring that data is handled in compliance with ethical guidelines and regulatory standards is crucial for fostering trust and facilitating the broader adoption of XAI in medical CL neurotechnology [210]. Addressing these concerns will be vital for the future development of AI-enabled and complex algorithm-based neurotechnological systems [47].

At the software performance level, the trade-off between explainability and performance in AI-enabled medical CL neurotechnologies presents a critical challenge for their clinical adoption. High-performing models, such as DL and support vector machines (SVM), often deliver superior accuracy and predictive power, making them indispensable for complex applications like adaptive BCIs or DBS for psychiatric disorders. However, their opacity—the so-called ‘black-box’ nature—limits interpretability, raising concerns about transparency, accountability, and clinical trust. On the other hand, inherently interpretable models, such as LDA or decision trees, offer greater transparency but sometimes lack the sophistication needed to tackle more nuanced or high-dimensional data. While explainability is crucial for fostering clinician and patient trust, enhancing model transparency could compromise the system’s ability to deliver optimal therapeutic outcomes. This trade-off underscores the need for hybrid approaches, where interpretable components are integrated into high-performing models or augmented with XAI methods. Recent research highlights the significant potential of applying XAI to neurorehabilitation. For instance, explainable models have facilitated the identification of EEG biomarkers relevant to motor learning [211] and improved the prediction of upper limb rehabilitation outcomes in stroke patients by elucidating patient-specific predictive features [212]. Future efforts should focus on strategies that balance model performance with interpretability to ensure neurotechnologies are not only clinically effective but also transparent and ethically sound.

Beyond technical barriers, another potential obstacle to the integration of XAI methods into medical CL neurotechnology lies in the differing expectations, priorities, and languages among key stakeholders. A recent study identified three key differences between clinicians’ and developers’ perspectives on XAI: opposing goals, differing sources of truth, and an exploration versus exploitation mindset [7].These differing perspectives translate to a fundamental challenge for XAI, with many current methods being intended for developers who want to improve their systems, but not being tailored to clinicians and other users [213]. Partially, this may also reflect that the understanding of ethical challenges involved in AI-based medical CL neurotechnology development might not be the same for developers and clinicians [214]. Also, it remains ambiguous what aspects of an AI and other complex algorithm should be explainable or interpretable, and to whom these explanations should be directed. For instance, the explanation needed for a clinician may differ to the one for a patient, due to their level of knowledge. The same applies to patient-facing explanations in general. Patients differ in both their technical understanding and health literacy. To facilitate clinical adoption and ensure patient benefits, explanations should convey the AI’s reasoning in plain language and be complemented by accessible visual aids [215].

The absence of standardized terminology distinguishing explainability, interpretability, and explicability presents a major barrier to advancing this field—particularly within the context of neurological and psychiatric conditions. Although several studies emphasize the need for deeper investigation into model explainability and interpretability [40, 43, 120, 184], there remains a foundational gap: the lack of consensus on what aspects should be explained, how they should be explained, and to whom. For example, some researchers refer to explainability in relation to biomarkers [69] or clinical symptoms, while others highlight the inherent opacity of therapeutic mechanisms, such as those underlying DBS [90]. Others call for greater transparency in AI model decisions [40]. This conceptual ambiguity is compounded by the fact that the brain itself remains only partially understood from a biomedical standpoint. The interdisciplinary nature of the field—spanning neuroscience, engineering, and ethics—further increases its complexity. Consequently, it is essential to establish realistic expectations about what AI and other complex algorithms can meaningfully explain in the realm of medical CL neurotechnology, and to tailor those explanations to the needs of diverse stakeholders, including clinicians, patients, and regulators.

Crucially, the applicability of specific XAI methods depends on both the type of data and the algorithmic architecture employed. Nevertheless, a common advantage across the XAI methods identified in the reviewed literature is their capacity to illuminate which input features are most relevant for a given prediction or classification. This added transparency is a key benefit for a range of stakeholders. For instance, developers can use XAI to audit and refine model behavior by inspecting learned patterns, while clinicians may gain new insights into disease mechanisms or therapeutic targets—insights that could support diagnosis, treatment planning, and decision-making. Future work should prioritize systematic identification of clinicians’, patients’, and regulators’ exploratory needs, as current XAI methods are largely developer-oriented and seldom translate directly into clinical practice [216]. Empirically grounded user requirements will be essential for designing explanation methods that support clinical reasoning, increase acceptance and adoption, and ultimately improve patient care.

As our findings indicate, enhancing the explainability of AI-enabled and complex algorithm-driven medical CL neurotechnologies could be instrumental in fostering greater trust in these systems [205]. Yet, despite the frequent reference to “trust” in the reviewed articles, there remains a lack of clarity regarding the precise mechanisms through which explainability tout court or specific XAI methods might increase clinicians’ confidence in the validity, accuracy, and reliability of AI outputs, particularly in the fields of neurology and psychiatry [3, 213]. Trust is a critical factor in the adoption of AI-based technologies, and future research should aim to determine which XAI methods are most effective at fostering trust among clinicians and other users of AI in medical CL neurotechnology. To avoid double standards, it is also important to ensure that the evaluation of AI systems is fair and consistent compared to other technological advancements, such as robotics. Trust should be built on a foundation of solid explainability, which will be crucial for the successful translation of AI into medical practice and for advancing CL neurotechnology applications in the clinical setting.

Our analysis highlights three critical areas for addressing the current research gaps in the application of XAI within medical CL neurotechnology: advancing explainability methods, integrating them with emerging hardware capabilities, and ensuring alignment with ethical and regulatory standards. First, there is a pressing need to develop and rigorously validate XAI techniques that enhance transparency without compromising performance. This includes advancing tools for visualizing and interpreting model outputs and tailoring explainability to the needs of specific user groups, such as clinicians and patients. Second, as emerging neurotechnologies evolve, future research should explore how improvements in hardware such as extended battery life, neuromorphic processors, and cloud-based computational resources can support the implementation of both AI and XAI in CL neurotechnologies. These developments may mitigate current technical constraints while raising new ethical challenges that must be proactively addressed. Third, our review underscores the notable scarcity of empirical impact assessments regarding explainability in this field. This gap reinforces the urgent need for robust governance frameworks to manage neural data and AI-driven decision-making in ways that are transparent, fair, accountable and ethically sound. AI holds transformative potential for neurotechnology, offering unprecedented advantages in speed, pattern recognition, and real-time decision-making. These capabilities are particularly vital in high-stakes clinical domains such as stroke rehabilitation or seizure prediction in epilepsy, where rapid and accurate outputs can dramatically improve patient safety and outcomes. In these settings, AI models enable advanced feature selection, optimize signal interpretation, and capitalize on widely available off-the-shelf architectures, accelerating innovation in neurotechnological interventions. However, the opacity of most high-performing models presents a significant challenge, particularly in neurological and psychiatric contexts where patient vulnerability is high and the underlying mechanisms of disease are often incompletely understood. Black-box models limit clinicians’ ability to independently validate results, obscure the basis of therapeutic recommendations, and restrict the provision of informed consent. This lack of transparency also complicates regulatory oversight and public accountability. Bridging this divide requires a dual strategy: first, advancing inherently interpretable models that can provide clinically relevant predictions without sacrificing reliability; and second, integrating robust XAI techniques into more complex architectures to render their decisions more understandable and traceable. For domains where real-time predictions are essential, such as epilepsy or stroke, this balance between accuracy and interpretability is not only desirable but necessary to ensure that neurotechnological tools remain ethically responsible, clinically actionable, and legally compliant.

In light of this, we propose that XAI should not be regarded merely as a beneficial technical feature but rather as an ethical and epistemological imperative. Explainability is vital to ensuring that AI-enabled systems in medical CL neurotechnology are not only performant but also understandable, transparent, and aligned with core values in medicine, including safety, accountability, and patient autonomy. Explainable AI methods can help bridge the gap between algorithmic complexity and human interpretability by offering insights into how models generate predictions and which input features drive clinical decisions. In doing so, they are likely to support trust, empower human oversight, and reduce epistemic opacity, particularly in high-stakes fields such as neurology and psychiatry. To ensure the safe and ethically grounded implementation of such systems, future research must prioritize the development and integration of interpretable models, whether used in tandem with black-box models or as stand-alone alternatives [217].

International regulatory frameworks increasingly require alignment with explainability desiderata and XAI methods in a manner that goes beyond mere internal model validation. A key example is the European Union’s Artificial Intelligence Act (EU AI Act), which introduces a binding, risk-based framework for the development and use of AI systems across the EU. According to the Act, AI-enabled medical CL neurotechnology systems are likely to fall under the “high-risk” category due to their use in clinical decision-making and patient treatment, hence must adhere to strict transparency and explainability standards [5]. Specifically, Article 13, mandates that deployers of high-risk AI systems provide concise, clear, and understandable documentation on the system’s functionality, including the logic of its decision-making processes, the extent of human oversight, and foreseeable risks associated with the AI use [5]. This suggests that explainability will need to be operationalized not only for researchers and developers but also for clinicians, patients, and regulatory authorities. However, because harmonized standards, conformity practices, and official guidance for Article 13 are not yet univocally defined, it remains uncertain how these transparency obligations will be implemented in practice. At present, any concrete technical requirements for XAI in clinical neurotechnology should therefore be viewed as provisional interpretations rather than established regulatory expectations. Further empirical and regulatory work is needed to produce harmonized standards as well as medicine-focused conformity and compliance practices within the AI Act framework. In practice, AI-enabled neurotechnologies must produce outputs that are understandable to healthcare professionals, enabling informed clinical judgment, and be auditable by regulators to ensure fairness, bias mitigation, and safety. Transparent decision pathways, traceable logic, and adequate documentation are necessary to ensure these systems meet the EU’s standards of fairness, non-discrimination, and accountability.

Outside the European Union, other countries have begun formalizing their approaches to AI governance, though with varying degrees of emphasis on explainability. China has introduced several domain-specific regulatory instruments, including the *Regulations on the Administration of Internet Information Service Recommendation Algorithms* [218], the *Administrative Provisions on Deep Synthesis in Internet-Based Information Services* [219], and the *Interim Measures for the Management of Generative Artificial Intelligence Services* [220], all of which prescribe obligations related to algorithmic transparency and user understanding. South Korea’s AI Basic Act, adopted in December 2024 and slated for implementation in 2026, outlines a comprehensive national strategy for trustworthy AI, with a clear focus on ethical deployment and explainability requirements across sectors, including healthcare [221]. By contrast, countries like the United States and the United Kingdom have adopted more fragmented approaches that rely on sectoral oversight bodies and domain-specific guidance. While these approaches may lack the unified coherence of the EU framework, they still include emerging standards for transparency and human oversight through agencies such as the US Food and Drug Administration (FDA), the US National Institute of Standards and Technology (NIST), and the UK Information Commissioner’s Office. In all these cases, however, the normative importance of explainability is increasingly acknowledged, even if the scope and mechanisms of enforcement differ [222].

These operationalization requirements are also reflected in emerging inter-governmental multi-level governance instruments from the United Nations Educational, Scientific and Cultural Organization (UNESCO), the Organisation for Economic Co-operation and Development (OECD), and the Council of Europe. UNESCO’s *Recommendation on the Ethics of Artificial Intelligence* (2021) represents the first global standard-setting instrument on AI ethics adopted by all 193 member states. It calls explicitly for explainability and intelligibility in AI systems, particularly in health-related applications, and emphasizes that AI technologies must be transparent and understandable to ensure accountability and empower human agency [223]. The Recommendation mandates that AI systems should include mechanisms that make their decision-making processes auditable and interpretable by those affected by their outputs. Similarly, the OECD’s *AI Principles*, adopted in 2019 and endorsed by over 40 countries, include explainability as a key tenet of trustworthy AI [224]. These principles assert that AI systems should be transparent and explainable, and that stakeholders should have meaningful insight into AI-based decisions. This requirement is tied to broader principles of robustness, fairness, and human-centered values, urging developers and policymakers to adopt governance mechanisms that promote interpretability across the AI lifecycle.

As global regulatory frameworks continue to evolve, systems designed with explainability-by-design principles will be better prepared for certification, cross-border deployment, and real-world integration. Explainable AI, therefore, plays a pivotal role in enabling responsible innovation, aligning neurotechnological advances with ethical imperatives, and safeguarding the rights and well-being of patients in clinical practice.

## Limitations

Our scoping review has several limitations related to both the search process and the analysis itself. First, we restricted our review to articles written in English and German. This language limitation could have excluded important research published in other languages, thus reducing the comprehensiveness of our findings. Second, despite efforts to minimize selection bias by querying five databases, we cannot rule out the possibility that relevant research exists in other databases that were not included in our search. Third, variations in terminology and the search logic of different databases could mean that our search strategy, while comprehensive, may not have captured all relevant studies. Fourth, the dynamic and rapidly evolving nature of the field poses a temporal limitation. Our review includes articles published up to 12 January 2025. Given the fast pace of advancements in AI and medical CL neurotechnology, it is likely that more recent studies have emerged since our cut-off date and are therefore not reflected in this review. Fifth, due to the conceptual ambiguity surrounding the term “explainability” and the diversity of its interpretations across disciplines, some relevant articles may have been excluded, particularly those that do not explicitly mention explainability, its common synonyms, or established XAI methodologies, even if they engage with related concepts in practice. Sixth, another possible limitation is that the cutoff date of 2010 might have excluded XAI neurotechnological applications developed prior to that year. To mitigate this risk, we examined the temporal distribution of XAI-related work to ensure theoretical saturation and confirm that earlier years would not contribute additional explainability studies. This assessment showed that XAI implementations in CL neurotechnology begin only after 2019, with no application found between 2010 and 2019. Therefore, the 2010 cutoff likely captures the full emergence of contemporary AI-enabled CL systems while avoiding unnecessary inclusion of periods with no XAI activity. Seventh, although we employed a rigorous reflexive thematic analysis, the coding process is inherently interpretative, and thus subject to potential biases. This interpretative nature may have resulted in the underrepresentation of certain themes or nuances. From a more general perspective, scoping review methods offer valuable insights by capturing the breadth of research in fast-evolving fields. However, they are not designed to assess methodological quality or effect sizes. Given that many CL neurotechnology studies rely on very small sample sizes, often fewer than ten participants, the resulting models are highly sensitive to individual-level variation; consequently, XAI outputs in this context are inherently fragile and should be interpreted with caution, a constraint that may also help explain the low adoption of XAI observed in our review. Future work should include meta-analytical studies that systematically evaluate the performance, reliability, and real-world applicability of XAI techniques in the context of medical CL neurotechnology. Finally, our review is intentionally limited to CL neurotechnologies used in clinical neurology and psychiatry. CL systems in adjacent medical domains (e.g., pain, sleep, ICU monitoring, or general rehabilitation without neural interfacing) or in the consumer sector were not included to maintain conceptual coherence and align with scoping-review best practices for clearly defined boundaries. Future work should conduct similar assessments in other domains of CL technology application.

## Conclusion

While there is broad consensus on the necessity of explainability for AI in medical devices, the integration of XAI in current research on AI-enabled and complex algorithm-based medical CL neurotechnology appears to be minimal. This gap underscores the urgent need for further research into XAI within the context of medical CL neurotechnology for neurological and psychiatric conditions. Gathering specific user requirements from clinicians, patients, and regulators in the future is crucial to increase clinical acceptance and thus adoption rate (see [216]). Such research efforts are critical to advancing clinical understanding, bridging the divide between theoretical research and clinical applications, and ultimately enhancing outcomes for patients. A relevant aspect of this discourse is addressing the double standards often present in medical research, such as the varied expectations for explainability between medication mechanisms and AI-enabled systems. Emphasizing the application of interpretable models wherever technically feasible, regardless of the specific medical CL neurotechnology context, will be essential for fostering trust, transparency, and accountability [225]. Advances in medical CL neurotechnology are expected to generate insights that can inform regulators and contribute to the development of more comprehensive and practical standards and guidelines for its responsible implementation. Addressing these issues will be crucial for ensuring that AI-based medical CL neurotechnologies are both effective and trustworthy, ultimately benefiting patient care and advancing the integration of AI into medical practice.

## Data Availability

Not applicable.
